# Systematic review and meta-analysis on the therapeutic reference range for escitalopram: Blood concentrations, clinical effects and serotonin transporter occupancy

**DOI:** 10.3389/fpsyt.2022.972141

**Published:** 2022-10-17

**Authors:** Luzie Eichentopf, Christoph Hiemke, Andreas Conca, Jan Engelmann, Manfred Gerlach, Ursula Havemann-Reinecke, Gudrun Hefner, Vincenzo Florio, Maxim Kuzin, Klaus Lieb, Margareta Reis, Thomas G. Riemer, Alessandro Serretti, Georgios Schoretsanitis, Gerald Zernig, Gerhard Gründer, Xenia M. Hart

**Affiliations:** ^1^Department of Molecular Neuroimaging, Medical Faculty Mannheim, Central Institute of Mental Health, Heidelberg University, Mannheim, Germany; ^2^Department of Psychiatry and Psychotherapy, Institute of Clinical Chemistry and Laboratory Medicine, University Medical Center of Mainz, Mainz, Germany; ^3^Arbeitsgemeinschaft für Neuropsychopharmakologie und Pharmakopsychiatrie (AGNP)-Work Group “Therapeutic Drug Monitoring”, Nürnberg, Germany; ^4^Department of Psychiatry, Central Hospital, Sanitary Agency of South Tyrol, Bolzano, Italy; ^5^Department of Psychiatry and Psychotherapy, Johannes Gutenberg University Medical Center Mainz, Mainz, Germany; ^6^Department of Child and Adolescent Psychiatry, Psychosomatics and Psychotherapy, University Hospital Würzburg, Würzburg, Germany; ^7^Department of Psychiatry and Psychosomatics, University of Göttingen, Göttingen, Germany; ^8^Vitos Clinic for Forensic Psychiatry, Forensic Psychiatry, Eltville, Germany; ^9^Department of Psychiatry, Comprensorio Sanitario di Bolzano, Bolzano, Italy; ^10^Clienia Schlössli AG, Psychiatric and Psychotherapeutic Private Clinic, Academic Teaching Hospital of the University of Zurich, Oetwil am See, Switzerland; ^11^Department of Psychiatry and Psychotherapy, University Medical Center Mainz, Mainz, Germany; ^12^Department of Biomedical and Clinical Sciences, Linköping University, Linköping, Sweden; ^13^Department of Clinical Chemistry and Pharmacology, Skåne University Hospital, Lund, Sweden; ^14^Charité – Universitätsmedizin Berlin, Corporate Member of Freie Universität Berlin and Humboldt-Universität zu Berlin, Institute of Clinical Pharmacology and Toxicology, Berlin, Germany; ^15^Department of Biomedical and Neuromotor Sciences, University of Bologna, Bologna, Italy; ^16^Department of Psychiatry, Psychotherapy and Psychosomatics, Hospital of Psychiatry, University of Zurich, Zurich, Switzerland; ^17^Department of Psychiatry, Behavioral Health Pavilion, Northwell Health, The Zucker Hillside Hospital, Glen Oaks, NY, United States; ^18^Department of Psychiatry, Zucker School of Medicine at Northwell/Hofstra, Hempstead, NY, United States; ^19^Department of Pharmacology, Medical University of Innsbruck, Innsbruck, Austria; ^20^Private Practice for Psychotherapy and Court-Certified Witness, Hall in Tirol, Austria

**Keywords:** escitalopram, reference range, blood level, therapeutic drug monitoring, antidepressant response, clinical effects, adverse drug reaction, SERT occupancy

## Abstract

**Introduction:**

A titration within a certain therapeutic reference range presupposes a relationship between the blood concentration and the therapeutic effect of a drug. However, this has not been systematically investigated for escitalopram. Furthermore, the recommended reference range disagrees with mean steady state concentrations (11–21 ng/ml) that are expected under the approved dose range (10–20 mg/day). This work systematically investigated the relationships between escitalopram dose, blood levels, clinical effects, and serotonin transporter occupancy.

**Methods:**

Following our previously published methodology, relevant articles were systematically searched and reviewed for escitalopram.

**Results:**

Of 1,032 articles screened, a total of 30 studies met the eligibility criteria. The included studies investigated escitalopram blood levels in relationship to clinical effects (9 studies) or moderating factors on escitalopram metabolism (12 studies) or serotonin transporter occupancy (9 studies). Overall, the evidence for an escitalopram concentration/effect relationship is low (level C).

**Conclusion:**

Based on our findings, we propose a target range of 20–40 ng/ml for antidepressant efficacy of escitalopram. In maintenance treatment, therapeutic response is expected, when titrating patients above the lower limit. The lower concentration threshold is strongly supported by findings from neuroimaging studies. The upper limit for escitalopram’s reference range rather reflects a therapeutic maximum than a tolerability threshold, since the incidence of side effects in general is low. Concentrations above 40 ng/ml should not necessarily result in dose reductions in case of good clinical efficacy and tolerability. Dose-related escitalopram concentrations in different trials were more than twice the expected concentrations from guideline reports.

**Systematic review registration:**

[https://www.crd.york.ac.uk/PROSPERO/display_record.php?RecordID=215873], identifier [CRD42020215873].

## Introduction

Among the antidepressant drug class of selective serotonin reuptake inhibitors (SSRIs), escitalopram (ESC), the active S-enantiomer of racemic citalopram, shows the highest serotonin transporter (SERT) selectivity (1–3). ESC is primarily approved for the treatment of major depressive disorder (MDD) and generalized anxiety disorder. To attain optimal remission, drug monitoring guided dosing for ESC is highly recommended within a concentration range between 15 and 80 ng/ml (4). A titration within a certain therapeutic reference range (TRR) presupposes a valid relationship between the blood concentration and the therapeutic effect of a drug. However, a concentration/effect relationship has not been systematically explored for ESC (5). Furthermore, the recommended TRR deviates with mean steady state concentrations of 11–21 ng/ml that are expected under the approved dose range of 10–20 mg/day (4). Positron emission tomography (PET) studies have shown that drug concentrations in blood correlate well with SERT occupancy. Evidence was given that a target engagement of 80% SERT occupancy corresponds to clinical efficacy (6). For objective assessment of a TRR for ESC, the aims of this review were to evaluate the association between ESC blood levels (BL) and clinical outcome or BL and SERT occupancy.

## Materials and methods

**FIGURE 1:**
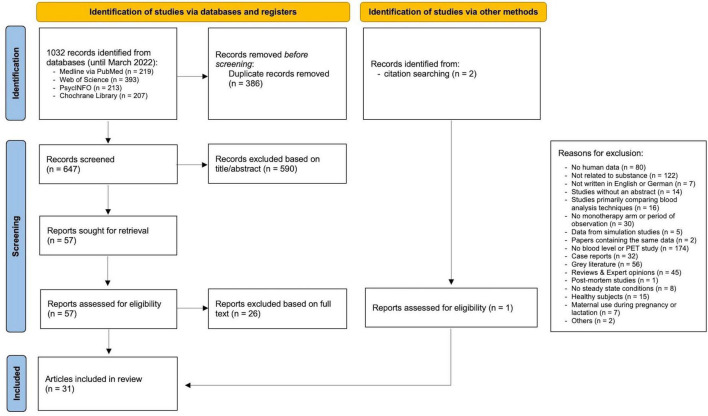
PRISMA flowchart of study selection. From Page et al. (8).

The systematic literature review was conducted following our previously published protocol (7) and relevant guidelines (8) including a quality control of studies and grading of available evidence (7). Four databases (MEDLINE via the PubMed interface, the Web of Science Core Collection, PsycINFO, and Cochrane Library) were screened using search terms for ESC, BL, therapeutic drug monitoring (TDM), PET and single-photon emission computed tomography (SPECT) (full search strings see Supplementary Table 1 in the Supplementary material). Our initial searches were carried out in October 2020 and were updated in March 2022 (for Preferred Reporting Items for Systematic Reviews and Meta-Analyses (PRISMA) Flowchart of study selection see Figure 1). Inclusion and exclusion criteria are presented in detail in the Supplementary material (Supplementary Table 2). Data extraction was performed according to our protocol (7). All steps were performed by two independent review authors (LE, XMH) and compared. We contacted authors of eligible trials for additional data, whenever concentration data was not complete. Our review protocol was registered on PROSPERO international prospective register of systematic reviews (CRD42020215873).

### Quality assessment of relevant studies and level of evidence

Six reviewers (LE, XMH, GH, MK, GG, MG) independently rated the quality of all included studies according to a previously reported rating instrument to assess the quality of TDM components of the studies and reporting (7). Two reviewers (TGR and XMH) rated the quality of the relevant efficacy cohort of randomized controlled clinical trials following the Cochrane risk-of-bias tool for randomized trials (RoB 2) (9). Any disagreements were resolved through discussion. Results were visualized using robvis (10). A level of evidence (7) for a concentration/effect relationship of ESC was found by consensus.

### Qualitative and quantitative synthesis

Reports were identified that examined an association between ESC and clinical outcome, either efficacy or side effects. These could be qualitative or quantitative, continuous or categorical, but required a structured clinical assessment using a rating scale. Factors that influenced ESC BL in patients were extracted. Studies that reported SERT occupancy in relation to ESC BL were extracted, and 80% effective concentrations (EC_80_ values) were calculated from the reported 50% effective concentrations (EC_50_ values). For quantitative synthesis, means, standard deviations (SDs), medians, and interquartile ranges (IQR) of the relevant BLs were assessed. Means and SDs of C/D ratios were selected. Data were either extracted from the manuscript or calculated manually when sufficient data was given.

### Statistical analysis

A combined meta-analysis was performed using the software R (Version 4.0.3) “metafor” and “meta” package. 95% confidence intervals (CIs) were calculated from mean concentrations and C/D values. Data was combined using random-effect models based on the I^2^ statistic. Four quality assessment criteria that could have a potential influence on the clinical validity of a reference range were identified a priory (Q2b “diagnosis depression,” Q3a “psychiatric comedication,” Q3b “cytochrome P450 (CYP) interfering comedication,” and Q4 “dose design”). Their impact as moderating factors on mean BLs were investigated by subgroup analyses of studies rated sufficient or insufficient on those criteria if a minimum of three records were available. Linear regression analysis was used to display the relationship between ESC dose and ESC BL.

### Study overview

For initially identified 647 articles excluding duplicates, 590 papers were rejected after reviewing title/abstract. Another 26 articles were excluded after full text screening, resulting in the inclusion of a total of 31 articles (11–41) (Figure 1), published between 2006 and 2021 (see Supplementary Table 3 for study details). Two articles reported results from the same study cohort (14, 15), resulting in a total of 30 studies. Most excluded studies did not report ESC blood concentrations, did not relate to substance, or did investigate non-human subjects. Included studies investigated ESC blood concentrations in relationship to clinical effects (9 studies), or moderating factors on ESC metabolism (12 studies), or SERT occupancy (9 studies).

### Risk of bias rating

General quality criteria for the TDM component were assessed for all 30 studies as shown in Supplementary Table 4 and Supplementary Figure 1. None of the studies was able to fulfill all TDM quality criteria. The most frequently missed criterion was “dose design” (Q4) due to flexible dosing in naturalistic settings. The second most frequently missed criterion was multiple concentration measurements (Q7a), whereas “sufficiently broad concentration range” (Q7b) was sufficient in most studies. “Comedication” (Q3) was rated as insufficient in 14 studies, mostly because of missing information. Most studies were retrospective in nature and included a heterogeneous patient sample according to “diagnosis” (Q2b). The majority of concentration/metabolism studies failed to select patients according to psychiatric classifications and the associated classification system, whereas the other study types usually did (Q2a). “Representativeness of the patient sample” (Q1) was not met by reason of healthy subjects in most of the included neuroimaging studies. Due to patient selection based on a genotype database, this criterion (Q1) was also not met in half of the concentration/metabolism studies, while most of the concentration-effect studies were able to meet. The analytical method (Q5) was rated as insufficient in 9 studies because description was insufficient or no validated analytical method was used. Sampling time (Q6b) and steady state (Q6a) were reported in the majority of selected studies. Study type specific quality assessment scores (ST scores) for cohort studies ranged from 3 to 10 (with a maximum score of 10), for cross sectional studies from 4 to 7 (with a maximum score of 8) and randomized controlled studies were assessed with some concerns to high risk of bias (results shown in Supplementary Tables 5–7 and Supplementary Figure 2).

## Results

### ESC concentration/efficacy relationship

Eight studies measured disease severity using psychiatric rating scales (11–14, 16–19). Out of these, two were open-label, randomized controlled trials, one was an observer-blinded randomized controlled trial, four were prospective cohort studies, and one was a cross-sectional study. Two studies did not report or investigate a concentration/effect relationship (16, 18). From the six remaining studies, a correlation between ESC blood concentration and response was only demonstrated by two studies of moderate (11) and high (14) risk for bias. A prospective cohort study by Florio et al. found larger improvement in Hamilton rating scale for depression version 21 (HAMD-21) from baseline with increasing ESC BLs as primary outcome after 3 months of constant dosing (study rating score of TDM component (TDM score) 9/10; ST score 5/10) (11). Notably, drug concentrations well below 10 ng/ml were preserved in their sample despite a flexible dosing design. The authors reported an optimal efficacy between 20 and 40 ng/ml, however, the use of a quadratic function to depict a concentration efficacy relationship has been criticized in the past (42). Using the original data, a threshold concentration was calculated that is able to separate responders from non-responders. The ROC curve (AUC 0.817) revealed a threshold concentration of 20.5 ng/ml (*p* < 0.05, Supplementary Figure 3). The second study by Hodgson et al. found a negative linear relationship between ESC blood concentration group defined by CYP2C19 genotypes and antidepressant response by Montgomery–Åsberg Depression Rating Scale (MADRS) at high ESC concentrations (TDM score 7/10; high risk) (14). However, when drug dose was included as a covariate in the analysis, this significant association between response to ESC treatment and serum concentrations of the drug disappeared (14). The four remaining studies failed to demonstrate a concentration-response relationship (12, 13, 17, 19). No systematic review or meta-analysis on the relationship between ESC blood levels and therapeutic response were available. As a result, the level of evidence for the relationship between ESC blood concentration and response is “low” (Level C).

### ESC concentration/side effect relationship

Five studies were identified that evaluated side effects during ESC treatment (Table 1). Of these, two were prospective cohort studies, two were randomized, controlled trials, and one was a cross-sectional study. One study investigated patients after ESC discontinuation and was excluded from analysis (20). Of the remaining studies, one study of high risk for bias (TDM score 8/10, ST score high risk) has found the expected relationship between side effects and ESC blood level (15). As part of a 12-week open-label part-randomized study, Hodgson et al. reported a correlation between dry mouth and increasing ESC blood level after week 8 (15). However, the ESC blood level did not predict the total number of adverse drug reactions (ADRs) in this study. Kuo et al. demonstrated a correlation between ESC metabolic ratios and specific side effects (12). One study could not demonstrate a relationship between the frequency of ADRs and ESC blood level (13). Overall, the evidence for the concentration/side effect relationship is low to absent (dry mouth; level low C).

**TABLE 1:** Studies investigating efficacy and/or side effects of ESC treatment, sorted by relevance.

References	Study design and subjects	PD comed.*	ST score	TDM score	BL/antidepressant effect relationship	BL/side effect relationship	Comment
Florio et al. (11)	pCS with flexible doses (mean 15 mg/d); MDD; N: 70	No	5/10	9/10	+ (HAMD-21A)	N/A	Focus on BL/effect-relationship.
Hodgson et al. (14)	RCT with flexible doses (mean 16 mg/d); UD; N: 266 (2014), 340 (2015)	No	High risk	8/10	– at high BL/ Ø for dose-corrected BL (MADRS)	–dry mouth (ASEC)	Focus on genotyping.
Yasui-Furukori et al. (20)	pCS with fixed doses (mean 5 mg/d); ADS; N: 25	No	6/10	7/10	N/A	–(DESS)	No value for TRR (ADS was investigated).
Kuo et al. (12)	pCS with flexible doses (mean 10 mg/d); MDD; N: 158	No	8/10	9/10	Ø (HAM-D, HAM-A, CGI-S, CGI-I)	± dry mouth, fatigue, nausea (TESS)	Focus on genotyping. Correlation between CYP1A2 SNPs and ADRs. No direct correlation of ADRs with BL.
Tadic et al. (13)	RCT with fixed doses (mean 19 mg/d); MDD; N: 889	No	some concerns	8/10	Ø (HAMD-17)	Ø (ADR frequency)	
Leuchter et al. (17)	RCT with fixed doses (mean 10 mg/d); MDD; N: 73	No	high risk	6/10	Ø (HAMD-17)	N/A	

PD Comed., Concomitant psychotropic medication with antidepressant efficacy; QA, quality assessment; pCS, prospective cohort study; N/A, not available; Ø, not found; +, positive correlation; –, negative correlation; BL, blood level; TRR, therapeutic reference range; MDD, major depressive disorder; UD, unipolar depression; ADS, antidepressants discontinuation syndrome; N, Subjects treated with ESC, *except for benzodiazepines.

### ESC concentration/serotonin transporter occupancy relationship

Nine studies were identified that investigate ESC BL in relation to SERT occupancy in the human brain (33–41). Of these, five were prospective cohort studies and four were randomized, controlled trials. Four SPECT studies could not be further considered due to methodological limitations using non-selective radiotracers (36, 37, 40, 41). The remaining five PET studies suggest that there are significant relationships between SERT occupancy and both ESC dose and BL. Two PET studies did not report EC_50_ or EC_80_ values (34, 39). Two of three remaining studies used single doses in healthy volunteers (33, 35) whereas in one study steady-state concentrations were achieved in patients with depression (38). Despite differing dose regimens and patient samples, the estimated EC_80_ values from the remaining three PET studies are quite consistent with a range between 16 and 18 ng/ml (Table 2) (33, 35, 38). One of the neuroimaging studies investigated transporter occupancy in regard to clinical effects but was not able to demonstrate an association between treatment response and SERT binding or SERT occupancy after 3 weeks of ESC administration (38). Previous work has suggested the EC_80_ value being related to the onset of antidepressant effects (6).

**TABLE 2:** EC_80_ values from neuroimaging studies.

References	No of subjects (males in%)	Mean age ± SD (years), if not specified other	Indication	Country	Steady state	EC80 in ng/ml	Brain region
Arakawa et al. (33)	16 (50)	29.1 ± 4.6	HV	Japan	no	16.0	Thalamus
Kim et al. (35)	12 (100)	23.0 ± 2.7	HV	Korea	no	17.2* 11.6*	Putamen DRN
Lanzenberger et al. (38)	10 (40)	42.3 ± 7.8	MDD	Austria	yes	17.5**	Thalamus

*SPECT study (semiquantitative); **Data derived from Baldinger et al. (47). DRN, Dorsal raphe nucleus; HV, healthy volunteers; MDD, major depressive disorder.

### Factors influencing ESC blood level

Twelve studies were identified that reported ESC concentrations and the influence of potential moderating factors shown in Table 3 (21–32).

**TABLE 3:** Factors influencing ESC serum concentration (SC).

References	CYP2C19 genotype	Dose	Sex	Age	Body weight	Smoking	Comedication
Bråten et al. (25)	**X^1^**		–				
Jukić et al. (26)	**X^2^**						
Reis et al. (22)		X^6^	–	X^12^	–	–	–^21^
Reis et al. (21)		X^7^	X^9^	**X^13^**			
Rudberg et al. (27)	**X^3^**						
Rudberg et al. (28)	**X^4^**						
Scherf-Clavel et al. (23)		X^8^	X^9^	–^14^		X^20^	
Tsuchimine et al. (29)	X^5^	X^8^		X^15^	–^18^		
Unterecker et al. (30)					–^19^		
Unterecker et al. (31)			–^10^	–^16^			
Waade et al. (32)	X		**X^11^**	X^17^			
Warrings et al. (24)					–		

*X* = correlation found; – = no correlation found; blank = not reported; bold = clinically relevant.

**Genotype** (if not specified other decrease/increase of ESC SC compared to CYP2C19*1/*1 group).

^1^Compared to baseline (no carriers of CYP2C19Null, CYP2C19*17, CYP2C:TG haplotypes): 1x/2x CYP2C:TG -16.7%/–24.8%; 1x/2x CYP2C19*17 –13.9%/–17.2%; 1x/2x CYP2C19Null (non–functioning alleles CYP2C19*2, *3 or *4) + 47%/ + 150%.

^2^CYP2C19*2/*2: + 230%; CYP2C19*2/*1: + 60%; CYP2C19*2/*17: + 40%; CYP2C19*1/*17: –10%; CYP1C19*17/*17: –20%.

^3^Non–dose–corrected SC: CYP2C19*2/*1: + 150%.

^4^Dose–adjusted SC: CYP2C19*17/*17: –42%; CYP2C19*1/*17: –13%; CYP2C19*17/def(*2 or *3 alleles): + 30%; CYP2C19*1/def: + 90%; CYP2C19def/def: + 470%.

^5^Significant difference among genotype groups in the dose–corrected steady–state SC.

**Dose**

^6^Dose–concentration linearity.

^7^Lower C/D ratios with increasing doses.

^8^ESC SC significantly increased with dose.

**Sex**

^9^Higher ESC SC in women vs men.

^10^No significant difference between males and females in the mean dose–corrected ESC SC.

^11^ + 80% higher C/D ratio in women vs men.

**Age**

^12^Higher age correlated with > ESC dose–normalized SC.

^13^ + 91% increase of ESC SC in subjects > 65y compared to subjects < 65 years at 10 mg/d.

^14^Linear regression analysis showed no influence of age (*P* = 0.301).

^15^Analysis of covariance: CYP2C19 genotypes and age were correlated with the steady–state ESC SC.

^16^No significant difference between patients < 60 years and patients > 60 years regarding the mean dose–corrected ESC SC.

^17^+40% higher mean C/D ratio in patients > 65 years, than in patients < 40 years (not significant in CYP2C19 PM, significant in other subgroups of CYP2C19 phenotype).

**Body weight**

^18^Analysis of covariance showed that CYP2C19 genotypes and body weight were not correlated with steady–state ESC SC.

^19^Referring to dose–corrected SC.

**Smoking**

^20^–24% lower ESC SC in smokers compared to non-smokers.

**Comedication**

^21^Concomitant medication did not interact with ESC. Women taking oral contraceptives had < metabolic ratio compared to women of the same age.

#### CYP2C19 genotyping

Six studies showed an association between ESC BL and CYP2C19 metabolism (25–29, 32). Four studies showed an increase in ESC BL (dose-corrected and non-dose-corrected) of up to 90% and 470% in patients carrying heterozygous and homozygous non-functioning CYP2C19 alleles (either *2, *3, or *4 alleles), respectively (25–28).

#### Sex

Another six studies examined the effect of sex on ESC BL (21–23, 25, 31, 32). Three of them reported an effect of sex on ESC blood concentrations (21, 23, 32), while three did not (22, 25, 31). Two studies found a 9 and 40% higher ESC BL in women, respectively (21, 23). One study found an 80% higher C/D ratio in women vs. men (32).

#### Age

Six studies examined the effect of age on ESC BL (21–23, 29, 31, 32), four of which found an association (21, 22, 29, 32), while two did not (23, 31). In one study, older age correlated with higher ESC dose-normalized BL (22). Another study showed a 91% increase in ESC BL in subjects over 65 years of age compared to subjects under 65 years of age at 10 mg/day (21). One study compared the C/D ratio in elderly (>65 years) with younger (<65 years) patients, which was increased by an average of 40% in the elderly (not significant for CYP2C19 low metabolizers) (32). Analysis of covariance in one study showed a correlation of CYP2C19 genotypes and age with the steady-state ESC BL (29). A linear regression analysis from another study showed no influence of age on the ESC BL (23). Another study found no significant difference in mean dose-corrected ESC BL between patients over 60 and patients under 60 (31).

#### Body weight

Four studies found no effect of body weight on ESC BL (22, 24, 29, 30).

#### Smoking

One study found an effect of smoking on ESC metabolism (23), reporting a 24% lower ESC BL in smokers compared to non-smokers.

#### Comedication

One study examined concomitant medications (heterogeneous group of different central nervous and somatic drugs) that did not interact with ESC metabolism (22). However, this study showed that women taking oral contraceptives (different substances) had a lower metabolic ratio compared to women of the same age not using contraception (22).

### Dose/concentration relationship

Four TDM studies reported a linear correlation between ESC dose and BLs (21–24). Mean C/D ratios across four studies carried out in a naturalistic setting with multiple diagnoses ranged between 1.09 and 2.47 (ng/ml)/(mg/day) (Table 4). Linear regression analysis showed that mean doses did not correlate with mean concentrations across 12 studies (*p* = 0.07) (data not shown).

**TABLE 4:** Expected ESC concentration [ng/ml] based on combined mean C/D ratio.

Author, year	Reference group	Dx	*n*	Mean	Combined mean C/D ratio*	5 mg/day	10 mg/day	15 mg/day	20 mg/day	25 mg/day
Reis et al. (22)	Somatically not healthy	mDx	45	1.67	1.23	*6*	*12*	*18*	*25*	*31*
	somatically healthy		89	1.00						
Reis et al. (21)	10 mg dose	mDx	1470	1.14	1.09	5**	11**	16**	22**	27**
	20 mg dose		883	1.00						
Scherf-Clavel et al. (23)	Smokers	mDx	36	1.69	2.22	11**	22**	33**	44**	56**
	Non-smokers		88	2.44						
Warrings et al. (24)	in- and outpatients	mDx	104	2.47	2.47	12**	25**	37**	49**	62**
Hiemke et al. (4)	young, male and female, somatically healthy	mDx	NA		1.05	5**	11**	16**	21**	26**

Dx: diagnose; mDx: multiple psychiatric diagnosis; N: naturalistic study setting, * [ng/ml/mg/day]. **Expected ESC concentration [ng/ml] based on the combined mean C/D ratio at administered dose [mg/day].

### Population-based target concentration range for ESC

**FIGURE 2:**
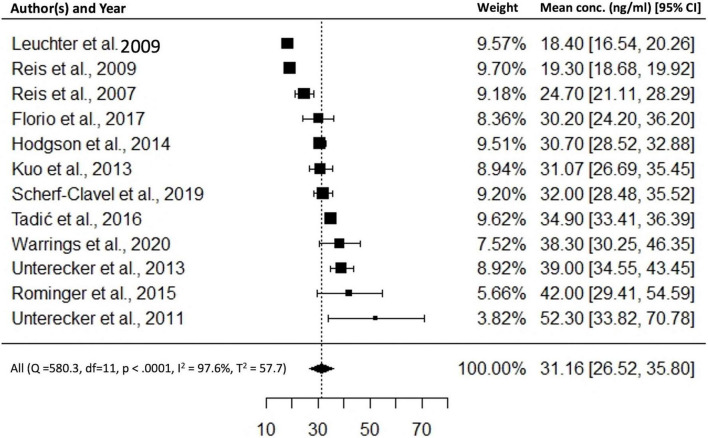
Combined mean ESC concentrations, *n* = 5,031.

**FIGURE 3:**
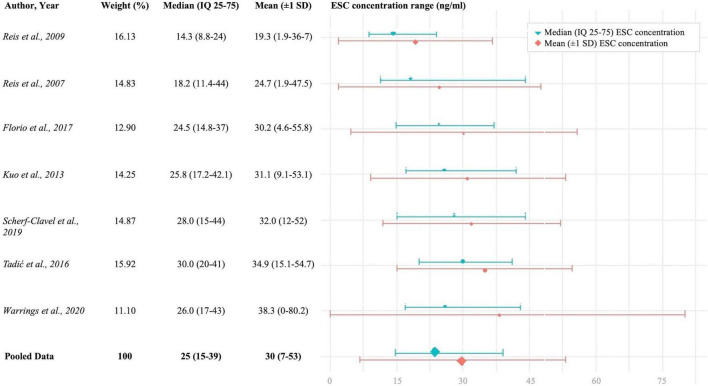
Combined ESC mean ± 1 SD and interquartile concentration ranges, *n* = 4,295.

Of 31 studies, 12 studies could be included in a quantitative synthesis (11–14, 17, 21–24, 30, 31, 40). 19 studies were excluded due to (i) antidepressant discontinuation, (ii) diagnosis other than depression, (iii) insufficient data reports, (iv) patients from genotype databases or genotype studies, (v) healthy sample cohort, (vi) same subject collective, and (vii) sampling at peak level. Across 12 studies a mean dose of 16 mg/day resulted in a combined mean ESC concentration of 31 (27, 36) ng/ml (*n* = 5,031, *Q* = 580.3, *p* < 0.0001, *I*^2^ = 97.6%, *T*^2^ = 57.7) (*p* ≤ 0.05, 95% confidence interval, Figure 2). Subgroup analysis could be performed with three predefined quality assessment criteria, since at least three studies per subgroup were available. Subgroup analysis for “dose design” could not be performed due to insufficient number of studies. Subgroup comparisons of “diagnosis depression,” “psychiatric comedication,” and “CYP interfering comedication” did show no significantly different mean drug concentrations between groups. Across 7 studies (11–13, 21–24), combined median ESC BL was 23.7 with an interquartile range of 15 and 39 ng/ml (*n* = 4,295, *Q* = 434.1, *p* < 0.0001, *I*^2^ = 96.6%, *T*^2^ = 37.0) (Figure 3). Two studies reported median and interquartile blood levels from responders treated with oral ESC under flexible dosing (11, 13). Interquartile ranges (median) of responders were 21–44 (29) ng/ml (*n* = 360), and 24–54 (36) ng/ml (*n* = 34), respectively.

## Discussion

The present work systematically explored concentration efficacy assumptions for the antidepressant drug escitalopram following a guideline-like methodology. Evidence for concentration/efficacy relationships of psychotropic drugs are in general low. This holds also true for the antidepressant drug escitalopram. None of the studies included in the present review examined a therapeutic reference range for ESC.

Our findings revealed an IQR (25–75%) of ESC concentrations across seven studies (*n* = 4,295) that is 15–39 ng/ml (trough levels 24 h after drug intake). While reporting a similar IQR range in the total sample (15–37 ng/ml), one study reported an IQR for responders of 24–54 ng/ml (11). Corresponding ROC analyses using 70 patients with depression of the beforementioned study (11) revealed a threshold concentration of 20.5 ng/ml for antidepressant response. Based on these findings, we suggest a lower threshold of 20 ng/ml for ESCs’ therapeutic reference range. Imaging studies consistently show that concentrations above 17 ng/ml will result in an adequate SERT occupancy above 80% (33, 35, 38).

For the upper threshold of ESCs’ reference range, we report a concentration threshold of 40 ng/ml that rather reflects an optimum in antidepressant response than decreased tolerability. 75% of all patients included in our metaanalysis showed drug concentrations below this threshold. As escitalopram is well tolerated with blood levels exceeding 40 ng/ml, serum concentrations above the upper threshold do not require a dose reduction in case of good clinical response and tolerability. Side effects seem not to limit ESC drug treatment, although the effect of increasing ESC BL on corrected QT (QTc) interval is controversially pointed out (43, 44). To sum up, we suggest a target range between 20 and 40 ng/ml for ESC drug monitoring during maintenance therapy (lower limit corresponds to 50% HAMD-21 improvement after 3 months with stable clinical effects).

The interpretation of highly varying dose-related concentration ranges raises questions. Firstly, dose corrected concentrations widely vary among studies (Table 4). A clear relationship cannot be established, not least due to highly varying sample compositions in terms of ethnicity, CYP2C19 polymorphisms, age and sex (Supplementary Table 8). This gives a strong indication for TDM of ESC, especially during dosing phases. An evident gap exists between real world dose-corrected concentrations and guideline reports (up to 2.47 vs. 1.05). Thus, at least in real world settings, ESC concentrations seem to vary strongly, but in general mean BLs will lie within the recommended efficacy range suggested in this work. Dose adaption might be required in elderly persons, in women, and in CYP2C19 poor metabolizers (Table 3).

A suggested reference range is strongly limited by the quality of the underlying study design that highly varied among studies. The presented information was mostly extracted from naturalistic TDM studies or small non-controlled studies. Since blood concentration measures are not prerequisite in drug approval, information from large pharmaceutical sponsored clinical trials were lacking. High quality randomized controlled trials investigating concentration efficacy relationships are missing as well as studies using a placebo lead-in phase. The inclusion of placebo responders might result in artificial concentration/effect relationships (14, 15) and in general altered mean and median BLs, expectedly toward lower values. Three studies comprising solely genotyped patients showed untypically low or high mean ESC BL and were therefore excluded from quantitative synthesis.

The present work aimed at providing a comprehensive overview of the efficacy and safety of ESC with regard to blood concentration ranges. As our primary focus is a concentration/effect relationship, we did not consider dose/response studies although these could have been provided additional insights, especially in regard to the upper limit of ESC, which might rather be related to a ceiling effect than to drug safety. There have been regulatory warnings for both citalopram and escitalopram on QT prolongation regardless of CYP2C19 phenotype (45). Studies investigating QT prolongation and incidence of QT prolongation/ADR (43, 44, 46) might in the future be considered to establish a laboratory alert level.

## Conclusion

Based on our results, we suggest a target range of 20–40 ng/ml for ESC’s antidepressant efficacy. The lower level hereby indicates a threshold for antidepressant response in maintenance therapy that is strongly supported by neuroimaging findings. Since the incidence of adverse drug reactions in ESC-treated patients was low to not quantifiable, the upper level of ESC is most likely best described by a maximum in clinical response (ceiling effect). A titration in concentration ranges above 40 ng/ml will most likely not result in a further increase in response in patients with insufficient response. However, concentrations above this range seem in general safe and should not lead to dose reductions in case of good response and tolerability.

Dose-related ESC concentrations measured in the different trials were more than twice the concentrations that would be expected for the given doses (4). Further clinical trials are needed to clarify this inconsistency.

## Data availability statement

The original contributions presented in this study are included in the article/Supplementary material, further inquiries can be directed to the corresponding author.

## Author contributions

LE developed the first draft of the protocol. XMH and GG supervised the entire manuscript writing and contributed to the revision of the protocol. XMH, LE, and GG contributed to the development of the search strategy. XMH, LE, GG, GH, MK, MG, and TGR contributed to the quality assessment. LE, XMH, CH, GG, AC, JE, MG, UH-R, GH, VF, MK, KL, MR, TGR, AS, GS, and GZ confirmed grading of the level of revealed evidence. All authors have read and approved the final manuscript.
