# Supplementary material for: Systematic review and meta-analysis on the therapeutic reference range for escitalopram: Blood concentrations, clinical effects and serotonin transporter occupancy
Source: Front Psychiatry. 2022 Oct 17;13:972141. doi: 10.3389/fpsyt.2022.972141 (PMC9621321; doi:10.3389/fpsyt.2022.972141)
Supplement: Supplementary file 1 [file Data_Sheet_1.pdf]

Luzie Eichentopf
Central Institute of Mental Health, Mannheim
Luzie.Eichentopf@zi-mannheim.de

Supplementary Material

"Systematic review and meta-analysis on the therapeutic reference range for escitalopram: blood concentrations, clinical effects and SERT occupancy"

Figure S1. Quality assessment results for TDM component…………………………………….… 2
Figure S2. Risk of bias assessment of RCTs using the ROB-2 tool……………………………...... 2
Figure S3. ROC curve for 70 ESC patients with MD……………………………………..……..… 3
Table S1. Full database search strings…………………………………………………………...… 4
Table S2. Inclusion and exclusion criteria for study eligibility…….…………………………… 5
Table S3. Detailed information on all included trials…………………………………………… 6
Table S4. Quality assessment for TDM component for all studies………...……………………… 9
Table S5. Quality assessment for cohort studies………………………………………………... 10
Table S6. Quality assessment for cross sectional studies………………………………….…..... 10
Table S7. Quality assessment for randomized controlled trials……………………………………10
Table S8. Steady-state ESC blood concentrations from 12 studies……………………………... 11
List of abbreviations………………………………………………………………………….. 12
Full list of included studies/ publications……………………………………………………….. 15

Luzie Eichentopf
Central Institute of Mental Health, Mannheim
Luzie.Eichentopf@zi-mannheim.de

Figure S1. Quality assessment results for TDM component

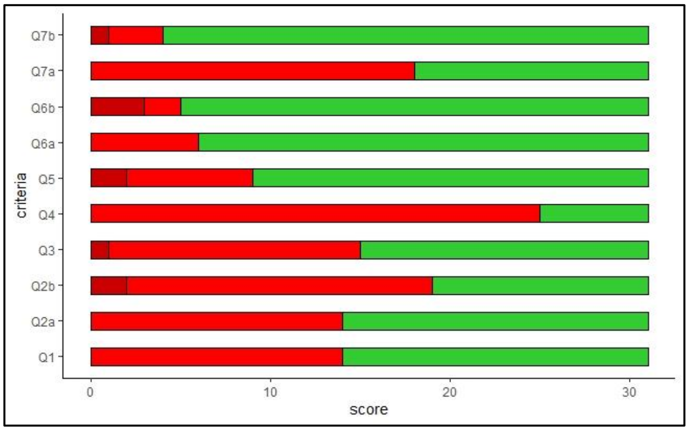

(Dark red, unclear; red insufficient; green, sufficient)

Figure S2. Risk of bias assessment of RCTs using the ROB-2 tool

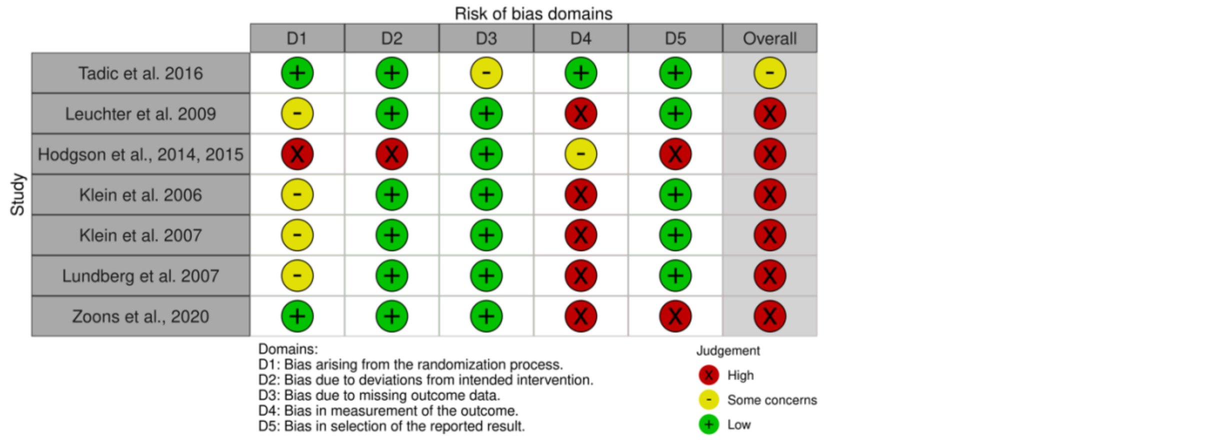

Traffic light plot of RCT bias assessment.

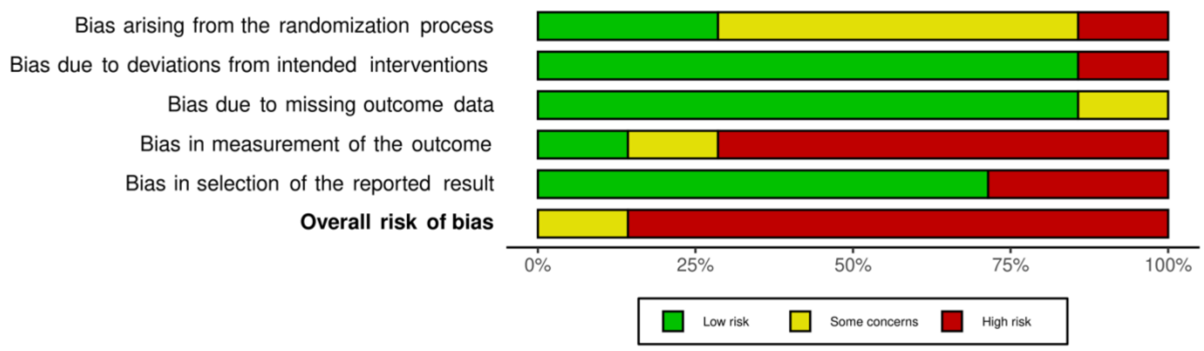

Weighted summary plot of the overall type of bias encountered in RCTs.

Luzie Eichentopf
Central Institute of Mental Health, Mannheim
Luzie.Eichentopf@zi-mannheim.de

Figure S3. ROC curve for 70 ESC patients with MD

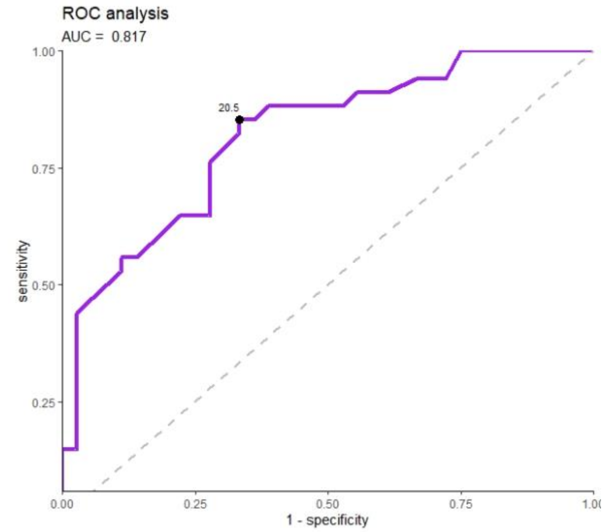

based on Florio et al.

Luzie Eichentopf
Central Institute of Mental Health, Mannheim
Luzie.Eichentopf@zi-mannheim.de

Table S1. Full database search strings

**PubMed**
("Escitalopram"[tw] OR "S Citalopram"[tw] OR "Cipralex"[tw] OR "Lexapro"[tw] OR "Seroplex"[tw]) AND ("serum level*"[tw] OR "plasma level*"[tw] OR "blood level*"[tw] OR "drug level*"[tw] OR "serum concentration*"[tw] OR "plasma concentration*"[tw] OR "blood concentration*"[tw] OR "drug concentration*"[tw] OR "Drug Monitoring"[Mesh] OR "drug monitor*"[tw] OR "positron emission tomography"[MeSH Terms] OR "Positron Emission Tomogra*"[tw] OR "PET scan*"[tw] OR "Tomography, Emission Computed, Single Photon"[Mesh] OR "Single Photon Emission*"[tw] OR "SPECT"[tw] OR "CAT Scan"[tw] OR "single photon emission computed tomography computed tomography"[MeSH Terms]) NOT ("Animals"[MeSH Terms] NOT "humans"[MeSH Terms])
**Web of Science Core Collection**
(TS=Escitalopram OR TS="S Citalopram" OR TS=Cipralex OR TS=Lexapro OR TS=Seroplex) AND (TS =(serum NEAR/1 level*) OR TS = (plasma NEAR/1 level*) OR TS= (blood NEAR/1 level*) OR TS=(drug NEAR/1 level*) OR TS=(serum NEAR/1 concentration*) OR TS=(plasma NEAR/1 concentration*) OR TS=(blood NEAR/1 concentration*) OR TS=(drug NEAR/1 concentration*) OR TS=(drug NEAR/1 monitor*) OR TS=(positron NEAR/1 emission NEAR/1 tomogra*) OR TS=(PET NEAR/1 scan*) OR TS=(single NEAR/1 photon NEAR/1 emission*) OR TS=SPECT OR TS=(CAT NEAR/1 Scan))
**Cochrane Library**
("Escitalopram" OR "S Citalopram" OR "Cipralex" OR "Lexapro" OR "Seroplex") AND ([mh "positron emission tomography"] OR [mh "Tomography, Emission-Computed, Single-Photon"] OR [mh "single photon emission computed tomography computed tomography"] OR (positron NEAR/1 emission NEAR/1 tomogra*) OR (PET NEAR/1 scan*) OR (tomography, emission NEAR/1 computed, single NEAR/1 photon) OR (single NEAR/1 photon NEAR/1 emission*) OR SPECT OR (CAT NEAR/1 Scan) OR (single NEAR/1 photon NEAR/1 emission) OR (single NEAR/1 photon NEAR/1 emission NEAR/1 computed NEAR/1 tomography NEAR/1 computed NEAR/1 tomograph*):ti,ab,kw OR (drug NEAR/1 monitor*):ti,ab,kw OR (serum NEAR/1 level*) OR (plasma NEAR/1 level*) OR (blood NEAR/1 level*) OR (drug NEAR/1 level*) OR (serum NEAR/1 concentration*) OR (plasma NEAR/1 concentration*) OR (blood NEAR/1 concentration*) OR (drug NEAR/1 concentration*)):ti,ab,kw
**PsycINFO**
("Escitalopram" OR "S Citalopram" OR "Cipralex" OR "Lexapro" OR "Seroplex") AND (MA "positron emission tomography" OR "positron emission tomogra*" OR "pet scan*" OR MA "tomography, emission computed, single photon" OR "single photon emission*" OR "SPECT" OR "CAT Scan" OR MA "single photon emission computed tomography computed tomography" OR MA "Drug Monitoring" OR "Drug Monitoring" OR "serum level*" OR "plasma level*" OR "blood level*" OR "drug level*" OR "serum concentration*" OR "plasma concentration*" OR "blood concentration*" OR "drug concentration*") NOT (MA "Animals" NOT MA "humans")

Luzie Eichentopf
Central Institute of Mental Health, Mannheim
Luzie.Eichentopf@zi-mannheim.de

Table S2. Inclusion and exclusion criteria for study eligibility

	Inclusion criteria	Exclusion criteria
Population	- Psychiatric patients treated with the ESC**<br>- Indication for ESC is MDD or anxiety disorders**<br>- For neuroimaging studies solely: healthy volunteers	- Non-human subjects<br>- Healthy volunteers**<br>- non-psychiatric patients**<br>- Post-mortem studies<br>- Maternal use during pregnancy or lactation
Intervention	- Oral ESC psychotropic monotherapy arm or period of observation (at least one ESC blood level measurement before add-on therapy)<br>- ESC blood levels in relation to dose in the steady state (6 days)**<br>- For neuroimaging studies solely: single dose studies	- ESC blood level is not measured in the steady state**<br>- Studies primarily comparing blood analysis techniques
Outcome(s)	- ESC concentrations measured in the blood (serum or plasma)<br>- For concentration/effect studies solely: direct clinical outcome measures are reported, i.e., safety or efficacy using a standardized rating scale (e.g., HAMD, MADRS, CGI)*<br>- For neuroimaging studies solely: ESC blood concentrations in relation to SERT occupancy investigated	- No mean or median ESC blood level reported
Study design	- Observational and interventional studies<br>- Reviews & meta-analysis investigating a concentration/effect relationship for ESC	- Reviews & experts' opinions<br>- Gray literature<br>- Case reports & case series
Other	- Written in English or German	- Papers containing the same data<br>- No abstract available<br>- Data from simulation studies

*Biomarkers (e.g. QTc-time) are not regarded a direct clinical outcome measure. ** Not applicable for neuroimaging studies.

Luzie Eichentopf
Central Institute of Mental Health, Mannheim
Luzie.Eichentopf@zi-mannheim.de

Table S3. Detailed information on all included trials

Author, year	Country	Design	Subjects (* = estimated from original data)	Mean ESC Dose ± SD [mg/day]	Mean ESC BL ± SD (ng/ml), if not specified other *=converted from original	Median ESC BL (ng/ml) *=converted from original	Comment	TDM score	Study score
**Concentration/effect studies**									
Hodgson et al., 2014	European project	RCT with flexible doses	N = 266 (ESC), Indication: UD; Mean age: 42.24 y (range: 19–72 y)	16.44 ± 6.44	30.74 ± 18.11	NA	Higher ESC SC predicting poorer treatment response, no cutoff	7/10	high risk
Hodgson et al., 2015	European project	RCT with flexible doses	N = 340 (ESC), Indication: UD; Mean age: 42.24 y (range: 19–72 y)	NA	29.6 ± 19.0	NA	Excluded from meta-analysis (same study cohort as *Hodgson et al., 2014)*	7/10	high risk
Leuchter et al., 2009	USA	RCT with fixed doses	N = 73 (34.2 % males), Indication: MDD; Mean age: 42.7 ± 12.7 y	10	18.4±8.1<br>Responder: 17.6 ± 7.8<br>(Unit not reported)	NA		6/10	high risk
Tadić et al., 2016	Germany	RCT with flexible doses	N = 95 (45.3 % males), Indication: MDD; Mean age: 38.9 ± 12.2 y	19.1 ± 1.9	34.8 ± 17.2	30 (25-75 IQR: 20-41)	No value for TRR (early improvers excluded).	8/10	some concerns
Florio et al., 2017	Italy	Prospective CS with flexible doses	N = 70 (40 % males), Indication: MDD; Mean age: 46.2 ± 16.63 y	15.2 ± 5.1	30.2 ± 25.6	24.5 (25-75 IQR: 15-37)	Higher ESC SC predicting higher treatment response.	9/10	5/10
Ji et al., 2014	USA	Prospective CS with flexible doses	N = 303 (38 % males), Indication: MDD; Mean age: 41.4 y	NA (10mg adjusted)	24.3 (range: 1.6–119.7)		Excluded from meta-analysis (missing data).	7/10	6/10
Kuo et al., 2013	Taiwan	Prospective CS with flexible doses	N = 158 (18 % males), Indication: MDD	8 w: 11.03 ± 4.09	8 w: 31.07 ± 22.02*	8 w: (N=97) 25.8 (25-75 IQR: 17-42)*		9/10	8/10
Steen et al., 2015	Norway	Prospective CS with flexible doses	N = 95 (ESC), Indication: SCZ + BD; Median age: 29 y (IQR: 16 y)	NA	NA	12.7 (SCZ);<br>14.6 (BD)<br>(IQR: 10.4 (SCZ);<br>13.6 (BD))*	No value for TRR (steady state not reported). Excluded from meta-analysis (diagnosis).	3/10	5/10
Yasui-Furukori et al., 2016	Japan	Prospective CS with fixed doses	N = 25, Indication: ADS; Mean age: 35.5 y	5	–ADS: 14.2 ± 19.7<br>+ADS: 36.5 ± 29.4	NA	No value for TRR, as ADS was investigated. No trough level. Negative correlation found for ADS. Excluded from meta-analysis (diagnosis).	7/10	6/10
Lloret-Linares et al., 2018	Switzerland	CSS with flexible doses	N = 10 (ESC), Indication: mDx; Median age: 49 y (range: 22–70 y)	Median: 20	NA	22 (range: 7.4–86.0)	Excluded from meta-analysis (no mean concentrations reported).	5/10	4/8
**Concentration studies**									
Jukic et al., 2018	Norway	Retrospective CS with flexible doses	N = 2087 (38% males); Indication: mDx; Mean age: 47.7 y	12.6 ± 6.0 (Null/Null);<br>15.8 ± 9.3 (Null/*1);<br>14.5 ± 6.3 (Null/*17);<br>18.0 ± 11.4 (*1/*1);<br>18.1 ± 9.8 (*1/*17);<br>17.1 ± 9.7 (*17/*17)	(adjusted to 10 mg/d)<br>33.93* (Null/Null);<br>16.88* (Null/*1);<br>14.45* (Null/*17);<br>10.39* (*1/*1);<br>9.35* (*1/*17);<br>8.47* (*17/*17)	NA	CYP2C19 genotype had a substantial impact on exposure and therapeutic failure of ESC (measured by switching of antidepressant therapy).	3/10	3/10
Reis et al., 2007	Sweden	Prospective CS with flexible doses	N = 212 (32% males); Indication: mDx; Mean age: 50 y (range: 13–95 y)	Median: 20 (range: 5–40)	NA	19.8 (range: 2.3-159.7)*	Higher age was correlated with higher SC,<br>no gender-related concentration differences were found, women taking oral contraceptives showed a lower metabolic ratio compared with age-matched women.	5/10	6/10
Bråten et al., 2021	Norway	CSS with flexible doses	N = 875; Indication: mDx	NA	NA	NA	Excluded from meta-analysis (missing data).	6/10	4/8
Reis et al., 2009	Sweden	CSS with flexible doses	N = 3066 (27% males); Indication: mDx; Median age: 44 y (range: 13–100 y)	NA	NA	14.3 (10-90 IQR: 5.8-37.3)*	Women had significantly higher ESC SC than men.<br>Patients >65 y had higher SC. ESC had lower C/D ratio with increasing doses with steep decline with a decreased	4/10	5/8

Luzie Eichentopf
Central Institute of Mental Health, Mannheim
Luzie.Eichentopf@zi-mannheim.de

							concentration of 0.11 nmol/L/mg/d.		
Rudberg et al., 2006	Norway	CSS with flexible doses	N = 43 (28% males); Indication: mDx; Mean age: 38.6 y	20 ± 9 (EM); 22 ± 10 (HEM)	NA	34.74* (EM) 13.64* (HEM)	Metabolism of CIT and ESC is impaired in CYP2C19 HEMs. Higher absolute SC indicate that this is not compensated for by dose reductions in clinical practice. Excluded from meta-analysis (missing data).	5/10	5/8
Rudberg et al., 2008	Norway	CSS with flexible doses	N = 166 (39% males); Indication: mDx; Mean age: 40 y	16 ± 5 (*17/*17); 22 ± 18 (*1/*17); 21 ± 13 (*1/*1); 20 ± 11 (*17/def); 20 ± 11 (*1/def); 11 ± 4 (def/def)	(in nM/mg/d) 1.59 (*17/*17); 2.36 (*1/*17); 2.72 (*1/*1); 3.51 (*17/def); 5.10 (*1/def); 15.5 (def/def)	NA	Homozygous CYP2C19*17 genotype associated with lower ESC SC.	6/10	5/8
Scherf-Clavel et al., 2019	Germany	CSS with flexible doses	N = 124 (31% males); Indication: mDx; Mean age: 50 ± 21 y	16 ± 7	32 ± 20	28 (25-75 IQR: 15-44)	Smokers received higher doses of ESC but showed lower SC and lower C/D. Men and women did not differ in terms of dose, women showed higher SC and higher C/D than men; Patients > 65 y did not differ regarding SC, but doses were significantly lower and C/D were higher.	6/10	6/8
Tsuchimine et al., 2018	Japan	CSS with flexible doses	N = 412 (29 % males); Indication: MDD, Mean age: 43.1 ± 17.3 y	5 (n=110) 10 (n=113) 15 (n=71) 20 (n=118)	Data given in this group order: EM, HEM, PM 5mg: 18.2±18.3, 26.2±28.7, 35.8±26.4; 10mg: 41.8±40.8, 40.1±31.7, 66.1±49.4; 15mg: 43.5±33.4, 55.6±30.8, 79.3±44.2; 20mg: 61.3±48.5, 68.8±30.8, 82.9±40.3	NA	No differences in the steady-state ESC or S-DCIT SC in each dose group (5, 10, 15, 20 mg) among CYP2C19 genotype groups. CYP2C19 variants associated with steady-state ESC SC but are not associated with S-DCIT. Age, sex, and body weight showed significant effects of CYP2C19 genotypes on the dose-adjusted SC. Age correlated with ESC SC. CYP2C19 genotypes and sex correlated with MPR.	7/10	7/8
Unterecker et al., 2011	Germany	CSS with flexible doses	N = 19 (32 % males); Indication: mDx; Mean age: 52.6 ± 24.0 y	19.7 ± 8.2	52.3 ± 41.1 (range: 14–191)	NA	Dose-corrected SC did not significantly correlate with body weight.	5/10	5/8
Unterecker et al., 2013	Germany	CSS with flexible doses	N = 359 (39 % males); Indication: mDx; Mean age: 48 ± 18 y	17 ± 8	39 ± 43 (range: 4–586)	NA	No significant difference between males and females in mean dose-corrected SC; No significant difference was found between patients > 60 y and patients up to 60 y regarding the mean dose-corrected SC; No additive effect of gender and age on dose-corrected SC.	5/10	5/8
Waade et al., 2014	Norway	CSS with flexible doses	N = 541; Indication: mDx	Range: 2.5-120	Data given in this group order: <40, 40-65, >65 y (in nmol/L/mg/d) PM: 9.3, 9.4, 13.2; HEM 5.0, 4.8, 7.1; EM: 2.8, 2.5, 3.6 (range: PM: 7.2–12.1, 7.9–11.2, 9.5–18.4; HEM: 4.3–5.9, 4.2–5.6, 5.6–9.1; EM: 2.5–3.1, 2.3–2.8, 3.0–4.2)	NA	No genotype-related effect of age for mean dose-adjusted ESC SC. Among PMs >65 y, none had absolute ESC SC above the upper recommended concentration range. In comparison, 25% of PMs <40 y and aged 40–65 y had measured absolute ESC SC above the upper recommended concentration range. Gender was found to influence the C/D ratio of ESC in CYP2C19 PMs. Further, gender was found to significantly influence the metabolic	4/10	5/8

Luzie Eichentopf
Central Institute of Mental Health, Mannheim
Luzie.Eichentopf@zi-mannheim.de

							ratio ESC/ S-DCT in CYP2C19 PMs. Gender was found to significantly influence the metabolic ratio in CYP2C19 EMs.		
Warrings et al., 2020	Germany	CSS with flexible doses	N = 104 (43% males); Indication: mDx; Mean age: 52.2 ± 17.1 y	16.5 ± 8.6	38.3 ± 41.9 (range: 0-317)	26 (25-75 IQR: 17-43)	Concentration/dose was not associated with BMI and was not different in normal weight, overweight, and obese patients.	5/10	4/8
**Neuroimaging studies**									
Klein et al., 2007	Austria	RCT using SPECT ([123I]ADAM) with fixed doses	N = 15 (100% males); Indication: HV; Mean age: 28 ± 7 y	10	5.5h: 16.69*±4.19*; 53.5h: 4.61*±1.46*	NA	No value for TRR (SPECT (semiquantitative), EC80 cannot be calculated correctly).	6/10	high risk
Klein et al., 2006	Austria	RCT using SPECT ([123I]ADAM) with fixed single doses	N = 29 (100 % males); Indication: HV; Mean age: 26.8 ± 4.3 y	5, 10, 20	5mg: 4.06*±0.94*<br>10mg: 7.2*±1.3*<br>20mg: 14.16*±3.12*<br>(measured 330 min after application)	NA	No value for TRR (SPECT (semiquantitative), EC80 cannot be calculated correctly).	4/10	high risk
Lundberg et al., 2007	Sweden	RCT using PET ([11C]MADAM) with fixed single doses	N = 8 (100% males); Indication: HV; Age range: 22-33 y	10	1h: 4.58*±3.25*<br>2h: 7.76*±1.75*<br>3h: 8.12*±2.08*<br>4h: 8.12*±1.46*<br>6h: 7.01*±1.17*<br>6.5h: 6.62*±1.46*<br>7h: 6.59*±1.62*<br>7.5h: 6.53*±1.33*<br>8h: 6.56*±1.46*<br>12h: 6.04*±1.27*<br>24h: 4.35*±1.36*<br>48h: 2.63*±1.23*	NA	No value for TRR. EC50 values not reported. EC80 value cannot be derived from published data.	4/10	high risk
Zoons et al., 2020	Netherlands	RCT using SPECT ([123I]FP-CIT) with fixed doses	N = 16 (62.5% males); Indication: CD; Mean age: 56.6 y	10	NA	NA	No value for TRR (SPECT (semiquantitative), Tracer (FP-Cit) unselective for SERT, no EC50 reported)	7/10	high risk
Arakawa et al., 2016	Japan	CCT using PET ([11C]DASB) with fixed single doses	N = 16 (50% males); Indication: HV; Mean age: 29.1 ± 4.6 y	10, 20	10mg: 13.8 ± 2.3 (4h); 6.5 ± 1.8 (24h); 3.2 ± 1.2 (48h)<br>20mg: 25.3 ± 6.9 (4h); 11.3 ± 3.5 (24h); 4.9 ± 2.1 (48h	NA	EC50 values reported. EC80 value can be derived from published data, but single doses applied in 4 HV (EC50 4ng/ml). EC80nss = 16 ng/ml (thalamus) in HV.	5/10	8/10
Hjorth et al., 2021	Sweden	Cohort nested in RCT using PET ([11C]DASB + [11C]PE2I) with fixed doses	N = 27 (63 % males); Indication: SAD; Mean age: 31.1 ± 10.3 y	20	30±17.66*	NA	No value for TRR. EC50 values not reported. EC80 value cannot be derived from published data.	5/10	10/10
Kim et al., 2017	Korea	Single-blind CCT using PET ([11C]DASB) with fixed single doses	N = 12 (100% males); Indication: HV; Mean age: 23.0 ± 2.7 y	5, 10, 20, 30	Cmax:<br>5mg: 4.8±0.8; 10mg: 9.5±0.7; 20mg: 21.7; 30mg: 24.1±1.5	NA	EC80nss = 17.2 ng/ml (putamen) in HV, EC80nss = 11.56 ng/ml (DRN) in HV.	6/10	9/10
Lanzenberger et al., 2012	Austria	Prospective CS using PET ([11C]DASB) with fixed doses	N = 19 (32% males); Indication: MDD; Mean age: 42.3 ± 7.8 y	10	PET2: 6.92±2.25 (range: 1–12)<br>PET3: 14.76±6.02 (range: 6–26)	NA	EC50 values not reported. EC80 value for steady-state concentrations can be derived from Baldinger et al., 2014 (diagram). EC80ss = 17,5 ng/ml (thalamus).	9/10	10/10
Rominger et al., 2015	Germany	Prospective CS using SPECT ([123I]ß-CIT) with flexible doses	N = 19 (42% males); Indication: MDD; Mean age 44.1±15.8 y	14.2 ± 4.8	42±28	NA	No value for TRR (SPECT (semiquantitative), Tracer (ß-Cit) unselective for SERT, no EC50 reported)	6/10	6/10

Luzie Eichentopf
Central Institute of Mental Health, Mannheim
Luzie.Eichentopf@zi-mannheim.de

Table S4. Quality assessment for TDM component for all studies

No	Reference	Q1	Q2	Q3	Q4	Q5	Q6	Q7	TDM Score (X/10)	Comment Meta-Analysis
**Concentration/effect studies**										
1a	*Hodgson et al., 2014*	X	XX	-	-	X	XX	- X	7/10	I
1b	*Hodgson et al., 2015*	X	XX	-	-	X	XX	- X	7/10	E: same subject collective
2	*Leuchter et al., 2009*	X	XX	X	X	-	- X	- ?	6/10	I
3	*Tadić et al., 2016*	X	XX	X	-	X	XX	X -	8/10	I
4	*Florio et al., 2017*	X	XX	X	-	X	XX	XX	9/10	I
5	*Ji et al., 2014*	X	X ?	X	-	X	X ?	XX	7/10	E: missing data
6	*Kuo et al., 2013*	X	XX	X	-	X	XX	XX	9/10	I
7	*Steen et al., 2015*	-	- X	-	-	-	X ?	- X	3/10	E: due to Dx
8	*Yasui-Furukori et al., 2016*	-	X ?	X	X	X	XX	- X	7/10	E: due to scales
9	*Lloret-Linares et al., 2018*	X	X -	-	-	-	XX	- X	5/10	E: missing data
**Concentration studies**										
No	***Reference***	***Q1***	***Q2***	***Q3***	***Q4***	***Q5***	***Q6***	***Q7***	***TDM Score (X/10)***	***Comment Meta-Analysis***
10	*Jukic et al., 2018*	-	- -	-	-	-	XX	- X	3/10	E: missing data
11	*Reis et al., 2007*	X	- -	-	-	X	XX	- X	5/10	I
12	*Bråten et al., 2021*	-	- -	X	-	X	XX	XX	6/10	E: missing data
13	*Reis et al., 2009*	X	- -	-	-	X	- X	- X	4/10	I
14	*Rudberg et al., 2006*	-	- -	X	-	X	XX	- X	5/10	E: missing data
15	*Rudberg et al., 2008*	-	- -	X	-	X	XX	XX	6/10	E: genotype study
16	*Scherf-Clavel et al., 2019*	X	- -	X	-	X	XX	- X	6/10	I
17	*Tsuchimine et al., 2018*	-	XX	X	-	X	XX	- X	7/10	E: genotype study
18	*Unterecker et al., 2011*	X	- -	-	-	X	XX	- X	5/10	I
19	*Unterecker et al., 2013*	X	- -	-	-	X	XX	- X	5/10	I
20	*Waade et al., 2014*	-	- -	-	-	X	XX	- X	4/10	E: genotype study
21	*Warrings et al., 2020*	X	- -	-	-	X	XX	- X	5/10	I
**Neuroimaging studies**										
No	***Reference***	***Q1***	***Q2***	***Q3***	***Q4***	***Q5***	***Q6***	***Q7***	***TDM Score (X/10)***	***Comment Meta-Analysis***
22	*Klein et al., 2007*	-	X -	-	X	-	XX	XX	6/10	E: healthy
23	*Klein et al., 2006*	-	X -	-	-	-	- X	XX	4/10	E: healthy
24	*Lundberg et al., 2007*	-	- -	X	-	X	- X	X -	4/10	E: healthy
25	*Zoons et al., 2020*	-	XX	-	X	X	X -	XX	7/10	E: due to Dx
26	*Arakawa et al., 2016*	-	- -	X	-	X	- X	XX	5/10	E: healthy
27	*Hjorth et al., 2021*	X	XX	?	X	?	X ?	- -	5/10	E: missing data
28	*Kim et al., 2017*	-	X -	X	-	X	- X	XX	6/10	E: healthy
29	*Lanzenberger et al., 2012*	X	XX	X	X	?	XX	XX	9/10	E: peak level
30	*Rominger et al., 2015*	X	XX	X	-	-	X -	- X	6/10	I

X= sufficient; - = insufficient; ? = not clear; I: Included; E: Excluded

Luzie Eichentopf
Central Institute of Mental Health, Mannheim
Luzie.Eichentopf@zi-mannheim.de

Table S5. Quality assessment for cohort studies

No	Reference	Selection (Max. 4 p)				Comparability (Max 2p)	Outcome (Maximum 3 p)				Total score (x/10)
		Q1	Q2	Q3	Q4	Q5	Q6	Q7	Q8	Q9	
4	*Florio et al., 2017*	X	-	-	X	- -	X	X	X	-	5/10
5	*Ji et al., 2014*	X	X	-	X	XX	-	X	-	-	6/10
6	*Kuo et al., 2013*	X	-	X	X	X -	X	X	X	X	8/10
7	*Steen et al., 2015*	-	X	-	-	XX	X	-	-	X	5/10
8	*Yasui-Furukori et al., 2016*	-	-	X	X	X -	-	X	X	X	6/10
10	*Jukic et al., 2018*	-	-	-	-	- X	-	X	-	X	3/10
11	*Reis et al., 2007*	X	-	-	-	XX	X	-	X	X	6/10
26	*Arakawa et al., 2016*	-	X	X	X	X-	X	X	X	X	8/10
27	*Hjorth et al., 2021*	X	X	X	X	XX	X	X	X	X	10/10
28	*Kim et al., 2017*	-	X	X	X	XX	X	X	X	X	9/10
29	*Lanzenberger et al., 2012*	X	X	X	X	XX	X	X	X	X	10/10
30	*Rominger et al., 2015*	X	-	X	X	- -	-	X	X	X	6/10

X= sufficient, - = insufficient, ? = not clear

Table S6. Quality assessment for cross sectional studies

No	Reference	Selection (Max 4 p):				Comparability (Max 2 p):	Outcome (Max 2 p)		Total score (x/8)
		Q1	Q2	Q3	Q4	Q5	Q6	Q7	
9	*Lloret-Linares et al., 2018*	X	-	X	X	- -	-	X	4/8
12	*Bråten et al., 2021*	-	X	-	-	X -	X	X	4/8
13	*Reis et al., 2009*	X	-	-	-	XX	X	X	5/8
14	*Rudberg et al., 2006*	-	-	X	-	XX	X	X	5/8
15	*Rudberg et al., 2008*	-	-	X	-	XX	X	X	5/8
16	*Scherf-Clavel et al., 2019*	X	-	X	-	XX	X	X	6/8
17	*Tsuchimine et al., 2018*	X	-	X	X	XX	X	X	7/8
18	*Unterecker et al., 2011*	X	-	-	-	XX	X	X	5/8
19	*Unterecker et al., 2013*	X	-	-	-	XX	X	X	5/8
20	*Waade et al., 2014*	-	-	-	X	XX	X	X	5/8
21	*Warrings et al., 2020*	X	-	-	-	X -	X	X	4/8

X= sufficient, - = insufficient, ? = not clear

Table S7. Quality assessment for randomized controlled trials

No	Reference	RoB arising from the randomization process	RoB due to deviations from the intended interventions	RoB due to missing outcome data	RoB in measurement of the outcome	RoB in selection of the reported result	RoB for trial
3	*Tadic et al. 2016*	low risk	low risk	some concerns	low risk	low risk	some concerns
2	*Leuchter et al. 2009*	some concerns	low risk	low risk	high risk	low risk	high risk
1	*Hodgson et al., 2014, 2015*	high risk	high risk	low risk	some concerns	high risk	high risk
23	*Klein et al. 2006*	some concerns	low risk	low risk	high risk	low risk	high risk
22	*Klein et al. 2007*	some concerns	low risk	low risk	high risk	low risk	high risk
24	*Lundberg et al. 2007*	some concerns	low risk	low risk	high risk	low risk	high risk
25	*Zoons et al., 2020*	low risk	low risk	low risk	high risk	high risk	high risk

Luzie Eichentopf
Central Institute of Mental Health, Mannheim
Luzie.Eichentopf@zi-mannheim.de

Table S8. Steady-state ESC blood concentrations from 12 studies with representative patient sample (Q1)

Reference	Indication	Comed. incl. Psychiatric	Dosing	Trough level	Mean age	N (ESC) (males in %)	Weight (%) A, B	Mean dose ± SD (mg/d)	ESC concentrations (ng/mL) *converted from original data (nM to ng/ml (converting factor: 3.08))			Comment
									**Mean** ± SD	**Median**	**IQ25-IQ75**	
Hodgson et al., 2014	UD	Y	flexible	Y	42.24	266	A: 9.51	16 ± 6	31 ± 18	N/A	N/A	Outpatients
Leuchter et al.	MDD	N	fixed	N/A	42.7	73 (34.2)	A: 9.58	10	18 ± 8	N/A	N/A	In- and Outpatients, not acute, Unit not reported
- Responders					42.9	38		N/A	18 ± 8	N/A	N/A	ROC threshold: 58.6
Tadić et al., 2016	MDD	N	flexible	Y	40.6$_A$	679	A: 9.62<br>B: 15.92	19 ± 2	35 ± 20	30	20 - 41	Inpatients, Response week 4, early (2 w) ESC nonresponders excluded
-Responders (50%)					41.5$_A$	360		19 ± 2	34 ± 21	29	21 - 44	
Florio et al., 2017	MDD	N	flexible	Y	46.2	70	A: 8.36<br>B: 12.90	15 ± 5	30 ± 26	24.5	15 - 37	Outpatients
- Responders						34		18 ± 6	43 ± 30	36.0	24 -54	HAMD-21 improvement ≥ 50% after 3 months
Kuo et al., 2013	MDD	N	flexible	N	N/A	97	A: 8.94<br>B: 14.25	11 ± 4	31 ± 22*	25.8*	17 - 42*	Outpatients, Sampling 12–20 h after last dose
Rominger et al., 2015	MDD	N	flexible	PN	44.1	19 (42)	A: 5.66	14 ± 5	42 ± 28	N/A	N/A	PET study, sampling time NA
Reis et al., 2007	Multiple Dx	Y	flexible	N	50	155	A: 9.18<br>B: 14.83	18 ± 9	25 ± 23*	18.2*	11 - 44*	Smpling 10-30 h after drug intake
Reis et al., 2009	Multiple Dx	Y	flexible	PY	44	3066 (27)	A: 9.70<br>B: 16.13	15 ± 7	19 ± 17*	14.3*	9 – 24*	Trough not confirmed
Scherf-Clavel et al., 2019	Multiple Dx	Y	flexible	Y	50	124 (31)	A: 9.20<br>B: 14.87	16 ± 7	32 ± 20	28	15 - 44	Inpatients and outpatients
Unterecker et al., 2011	Multiple Dx	Y	flexible	Y	53	19 (32)	A: 3.82	20 ± 8	52 ± 41	N/A	N/A	
Unterecker et al., 2013	Multiple Dx	Y	flexible	Y	48	359 (39)	A: 8.92	17 ± 8	39 ± 43	N/A	N/A	
Warrings et al., 2020	Multiple Dx	Y	flexible	Y	52	104 (43.3)	A: 7.52<br>B: 11.10	17 ± 9	38 ± 42	26	17 - 43	Inpatients and outpatients
**Pooled data**					41.83	A 5031	A 100		**A 31.2**			
						B 4295	B 100			**B 29.7**	**B**	

Luzie Eichentopf
Central Institute of Mental Health, Mannheim
Luzie.Eichentopf@zi-mannheim.de

List of abbreviations

AD	Anxiety disorders
ADS	Antidepressants discontinuation syndrome
ASEC	Antidepressant Side Effect Checklist
BD	Bipolar Disorders
BDI	Beck Depression Inventory
BL	Blood level
BZD	Benzodiazepine use
C/D	Concentration-to-dose (mean C / mean D)
CCT	Controlled clinical trail
CD	Cervical dystonia
CF	Conversion factor
CGI	Clinical Global Impression
CGI-I	Clinical Global Impression- Improvement
CGI-S	Clinical Global Impression - Severity
CIT	Citalopram
Conc.	Concentration
CS	Cohort study
CSS	Cross-sectional Study
CVLT	California Verbal Learning Test
CYP	Cytochrome P450
D-KEFS	Delis–Kaplan Executive Function Scale
d	Day
DESS	Discontinuation-emergent signs and syndromes (DESS) checklist
DSM-5	Diagnostic and Statistical Manual of Mental Disorders, 5th edition
Dx	Diagnosis
EM	Homozygous extensive metabolizers
EOCD	Early-onset obsessive-compulsive disorder
ESC	Escitalopram
GAF-S	Global Assessment of Functioning, symptom scale
HAMA/HAM-A	Hamilton Rating Scale for Anxiety
HAMD/HAM-D	Hamilton rating scale for depression
HEM	Heterozygous extensive metabolizers

HPLC with UV detection	High-performance liquid chromatography method with UV-absorbance detection
HV	Healthy volunteers
ICD-10	International Statistical Classification of Diseases and Related Health Problems, 10th edition
KEFS	Delis–Kaplan Executive Function Scale
LC/MS/MS	Liquid chromatography/ tandem mass spectrometry
LOD	Limit of detection
LOQ	Limit of quantification
m	Month
MADRS	Montgomery–Åsberg Depression Rating Scale
MD	Major depression
MDD	Major depressive disorder
mDx	Multiple Diagnosis
MINI	Mini International Neuropsychiatric Interview
MMSE	Mini-Mental State Examination
MPR	Metabolite to parent ratio
MR	Metabolic ratios
MS/MS	Tandem mass spectrometry
NA	Not available
OCD	Obsessive–compulsive disorder
PANSS	Positive and Negative Syndrome Scale
PBO	Placebo
PD	Panic disorder
PD Comedication	Concomitant psychotropic medication with antidepressant efficacy
PM	Poor metabolizers
QA	Result of the study-type specific quality assessment
QIDS-SR16	Quick Inventory of Depressive Symptomatology-Self Rated version
RCT	Randomized controlled trial
S-DCT	S-desmethylcitalopram
S-DDCT	S-didesmethylcitalopram
SAD	Social anxiety disorder
SC	Serum concentrations
SCTER	Serum concentration-therapeutic effect relationship
SCZ	Schizophrenia

Luzie Eichentopf
Central Institute of Mental Health, Mannheim
Luzie.Eichentopf@zi-mannheim.de

SD	Standard deviation
ST score	Study type specific quality assessment score
TDM	Therapeutic Drug Monitoring
TDM score	Quality assessment of the Therapeutic Drug Monitoring component score
TESS	Treatment Emergent Signs and Symptoms scale
TFC	Achiral turbulent flow liquid chromatography
TRR	Therapeutic reference range
TS	Tourette syndrome
UD	Unipolar depression
UKU	UKU side effect rating scale
UPLC-MS/MS	Ultra-performance liquid chromatography–tandem mass spectrometry
w	Week
WAIS	Wechsler Adult Intelligence Scale

Luzie Eichentopf
Central Institute of Mental Health, Mannheim
Luzie.Eichentopf@zi-mannheim.de

Full list of included studies/ publications (1-31)

1. Florio V, Porcelli S, Saria A, Serretti A, Conca A. Escitalopram Plasma Levels and Antidepressant Response. *Eur Neuropsychopharmacol* (2017) 27(9):940-4. Epub 2017/06/27. doi: 10.1016/j.euroneuro.2017.06.009.
2. Hodgson K, Tansey K, Dernovsek MZ, Hauser J, Henigsberg N, Maier W, et al. Genetic Differences in Cytochrome P450 Enzymes and Antidepressant Treatment Response. *J Psychopharmacol* (2014) 28(2):133-41. Epub 2013/11/22. doi: 10.1177/0269881113512041.
3. Hodgson K, Tansey KE, Uher R, Dernovšek MZ, Mors O, Hauser J, et al. Exploring the Role of Drug-Metabolising Enzymes in Antidepressant Side Effects. *Psychopharmacology (Berl)* (2015) 232(14):2609-17. Epub 2015/03/13. doi: 10.1007/s00213-015-3898-x.
4. Ji Y, Schaid DJ, Desta Z, Kubo M, Batzler AJ, Snyder K, et al. Citalopram and Escitalopram Plasma Drug and Metabolite Concentrations: Genome-Wide Associations. *Br J Clin Pharmacol* (2014) 78(2):373-83. Epub 2014/02/18. doi: 10.1111/bcp.12348.
5. Kuo HW, Liu SC, Tsou HH, Liu SW, Lin KM, Lu SC, et al. Cyp1a2 Genetic Polymorphisms Are Associated with Early Antidepressant Escitalopram Metabolism and Adverse Reactions. *Pharmacogenomics* (2013) 14(10):1191-201. Epub 2013/07/19. doi: 10.2217/pgs.13.105.
6. Leuchter AF, Cook IA, Marangell LB, Gilmer WS, Burgoyne KS, Howland RH, et al. Comparative Effectiveness of Biomarkers and Clinical Indicators for Predicting Outcomes of Ssri Treatment in Major Depressive Disorder: Results of the Brite-Md Study. *Psychiatry Res* (2009) 169(2):124-31. Epub 2009/08/29. doi: 10.1016/j.psychres.2009.06.004.
7. Lloret-Linares C, Bosilkovska M, Daali Y, Gex-Fabry M, Heron K, Bancila V, et al. Phenotypic Assessment of Drug Metabolic Pathways and P-Glycoprotein in Patients Treated with Antidepressants in an Ambulatory Setting. *Journal of Clinical Psychiatry* (2018) 79(2). doi: 10.4088/JCP.16m11387.
8. Steen NE, Aas M, Simonsen C, Dieset I, Tesli M, Nerhus M, et al. Serum Level of Venlafaxine Is Associated with Better Memory in Psychotic Disorders. *Schizophr Res* (2015) 169(1-3):386-92. Epub 2015/10/31. doi: 10.1016/j.schres.2015.10.021.
9. Tadic A, Wachtlin D, Berger M, Braus DF, van Calker D, Dahmen N, et al. Randomized Controlled Study of Early Medication Change for Non-Improvers to Antidepressant Therapy in Major Depression--the Emc Trial. *Eur Neuropsychopharmacol* (2016) 26(4):705-16. Epub 2016/02/24. doi: 10.1016/j.euroneuro.2016.02.003.
10. Yasui-Furukori N, Hashimoto K, Tsuchimine S, Tomita T, Sugawara N, Ishioka M, et al. Characteristics of Escitalopram Discontinuation Syndrome: A Preliminary Study. *Clin Neuropharmacol* (2016) 39(3):125-7. Epub 2016/05/14. doi: 10.1097/wnf.0000000000000139.
11. Bråten LS, Haslemo T, Jukic MM, Ivanov M, Ingelman-Sundberg M, Molden E, et al. A Novel Cyp2c-Haplotype Associated with Ultrarapid Metabolism of Escitalopram. *Clin Pharmacol Ther* (2021) 110(3):786-93. Epub 2021/03/25. doi: 10.1002/cpt.2233.
12. Jukić MM, Haslemo T, Molden E, Ingelman-Sundberg M. Impact of Cyp2c19 Genotype on Escitalopram Exposure and Therapeutic Failure: A Retrospective Study Based on 2,087 Patients. *Am J Psychiatry* (2018) 175(5):463-70. Epub 2018/01/13. doi: 10.1176/appi.ajp.2017.17050550.
13. Reis M, Aamo T, Spigset O, Ahlner J. Serum Concentrations of Antidepressant Drugs in a Naturalistic Setting: Compilation Based on a Large Therapeutic Drug Monitoring Database.

Ther Drug Monit (2009) 31(1):42-56. Epub 2008/12/17. doi: 10.1097/FTD.0b013e31819114ea.
14. Reis M, Chermá MD, Carlsson B, Bengtsson F. Therapeutic Drug Monitoring of Escitalopram in an Outpatient Setting. *Ther Drug Monit* (2007) 29(6):758-66. Epub 2007/11/29. doi: 10.1097/FTD.0b013e31815b3f62.
15. Rudberg I, Hendset M, Uthus LH, Molden E, Refsum H. Heterozygous Mutation in Cyp2c19 Significantly Increases the Concentration/Dose Ratio of Racemic Citalopram and Escitalopram (S-Citalopram). *Ther Drug Monit* (2006) 28(1):102-5. Epub 2006/01/19. doi: 10.1097/01.ftd.0000189899.23931.76.
16. Rudberg I, Mohebi B, Hermann M, Refsum H, Molden E. Impact of the Ultrarapid Cyp2c19*17 Allele on Serum Concentration of Escitalopram in Psychiatric Patients. *Clin Pharmacol Ther* (2008) 83(2):322-7. Epub 2007/07/13. doi: 10.1038/sj.clpt.6100291.
17. Scherf-Clavel M, Deckert J, Menke A, Unterecker S. Smoking Is Associated with Lower Dose-Corrected Serum Concentrations of Escitalopram. *J Clin Psychopharmacol* (2019) 39(5):485-8. Epub 2019/07/26. doi: 10.1097/jcp.0000000000001080.
18. Tsuchimine S, Ochi S, Tajiri M, Suzuki Y, Sugawara N, Inoue Y, et al. Effects of Cytochrome P450 (Cyp) 2c19 Genotypes on Steady-State Plasma Concentrations of Escitalopram and Its Desmethyl Metabolite in Japanese Patients with Depression. *Ther Drug Monit* (2018) 40(3):356-61. Epub 2018/03/24. doi: 10.1097/ftd.0000000000000506.
19. Unterecker S, Deckert J, Pfuhlmann B. No Influence of Body Weight on Serum Levels of Antidepressants. *Ther Drug Monit* (2011) 33(6):730-4. Epub 2011/11/23. doi: 10.1097/FTD.0b013e318237b0fa.
20. Unterecker S, Riederer P, Proft F, Maloney J, Deckert J, Pfuhlmann B. Effects of Gender and Age on Serum Concentrations of Antidepressants under Naturalistic Conditions. *J Neural Transm (Vienna)* (2013) 120(8):1237-46. Epub 2012/12/21. doi: 10.1007/s00702-012-0952-2.
21. Waade RB, Hermann M, Moe HL, Molden E. Impact of Age on Serum Concentrations of Venlafaxine and Escitalopram in Different Cyp2d6 and Cyp2c19 Genotype Subgroups. *Eur J Clin Pharmacol* (2014) 70(8):933-40. Epub 2014/05/27. doi: 10.1007/s00228-014-1696-8.
22. Warrings B, Samanski L, Deckert J, Unterecker S, Scherf-Clavel M. Impact of Body Mass Index on Serum Concentrations of Antidepressants and Antipsychotics. *Ther Drug Monit* (2020). Epub 2020/09/11. doi: 10.1097/ftd.0000000000000812.
23. Arakawa R, Tateno A, Kim W, Sakayori T, Ogawa K, Okubo Y. Time-Course of Serotonin Transporter Occupancy by Single Dose of Three Ssris in Human Brain: A Positron Emission Tomography Study with [(11)C]Dasb. *Psychiatry Res Neuroimaging* (2016) 251:1-6. Epub 2016/04/16. doi: 10.1016/j.pscychresns.2016.03.006.
24. Hjorth OR, Frick A, Gingnell M, Hoppe JM, Faria V, Hultberg S, et al. Expectancy Effects on Serotonin and Dopamine Transporters During Ssri Treatment of Social Anxiety Disorder: A Randomized Clinical Trial. *Transl Psychiatry* (2021) 11(1):559. Epub 2021/11/05. doi: 10.1038/s41398-021-01682-3.
25. Kim E, Howes OD, Kim BH, Chon MW, Seo S, Turkheimer FE, et al. Regional Differences in Serotonin Transporter Occupancy by Escitalopram: An [(11)C]Dasb Pk-Pd Study. *Clin Pharmacokinet* (2017) 56(4):371-81. Epub 2016/08/26. doi: 10.1007/s40262-016-0444-x.
26. Klein N, Sacher J, Geiss-Granadia T, Attarbaschi T, Mossaheb N, Lanzenberger R, et al. In Vivo Imaging of Serotonin Transporter Occupancy by Means of Spect and [123i]Adam in Healthy Subjects Administered Different Doses of Escitalopram or Citalopram.

Psychopharmacology (Berl) (2006) 188(3):263-72. Epub 2006/09/07. doi: 10.1007/s00213-006-0486-0.

27. Klein N, Sacher J, Geiss-Granadia T, Mossaheb N, Attarbaschi T, Lanzenberger R, et al. Higher Serotonin Transporter Occupancy after Multiple Dose Administration of Escitalopram Compared to Citalopram: An [123i]Adam Spect Study. *Psychopharmacology (Berl)* (2007) 191(2):333-9. Epub 2007/01/20. doi: 10.1007/s00213-006-0666-y.
28. Lanzenberger R, Kranz GS, Haeusler D, Akimova E, Savli M, Hahn A, et al. Prediction of Ssri Treatment Response in Major Depression Based on Serotonin Transporter Interplay between Median Raphe Nucleus and Projection Areas. *Neuroimage* (2012) 63(2):874-81. Epub 2012/07/26. doi: 10.1016/j.neuroimage.2012.07.023.
29. Lundberg J, Christophersen JS, Petersen KB, Loft H, Halldin C, Farde L. Pet Measurement of Serotonin Transporter Occupancy: A Comparison of Escitalopram and Citalopram. *Int J Neuropsychopharmacol* (2007) 10(6):777-85. Epub 2007/01/05. doi: 10.1017/s1461145706007486.
30. Rominger A, Cumming P, Brendel M, Xiong G, Zach C, Karch S, et al. Altered Serotonin and Dopamine Transporter Availabilities in Brain of Depressed Patients Upon Treatment with Escitalopram: A [123 I]B-Cit Spect Study. *Eur Neuropsychopharmacol* (2015) 25(6):873-81. Epub 2015/03/31. doi: 10.1016/j.euroneuro.2014.12.010.
31. Zoons E, Tijssen MAJ, Dreissen YEM, Smit M, Booij J. The Effect of Escitalopram on Central Serotonergic and Dopaminergic Systems in Patients with Cervical Dystonia, and Its Relationship with Clinical Treatment Effects: A Double-Blind Placebo-Controlled Trial. *Biomolecules* (2020) 10(6). Epub 2020/06/12. doi: 10.3390/biom10060880.
